# A CLK1-KKT2 Signaling Pathway Regulating Kinetochore Assembly in Trypanosoma brucei

**DOI:** 10.1128/mBio.00687-21

**Published:** 2021-06-15

**Authors:** Manuel Saldivia, Adam J. M. Wollman, Juliana B. T. Carnielli, Nathaniel G. Jones, Mark C. Leake, Christopher Bower-Lepts, Srinivasa P. S. Rao, Jeremy C. Mottram

**Affiliations:** a York Biomedical Research Institute, Department of Biology, University of York, Heslington, United Kingdom; b Novartis Institute for Tropical Diseases, Emeryville, California, USA; c York Biomedical Research Institute, Department of Physics, University of York, Heslington, United Kingdom; Yale University School of Public Health

**Keywords:** *Trypanosoma*, cell signaling, kinetochore, mitosis, protein kinases

## Abstract

During mitosis, eukaryotic cells must duplicate and separate their chromosomes in a precise and timely manner. The apparatus responsible for this is the kinetochore, which is a large protein structure that links chromosomal DNA and spindle microtubules to facilitate chromosome alignment and segregation. The proteins that comprise the kinetochore in the protozoan parasite Trypanosoma brucei are divergent from yeast and mammals and comprise an inner kinetochore complex composed of 24 distinct proteins (KKT1 to KKT23, KKT25) that include four protein kinases, CLK1 (KKT10), CLK2 (KKT19), KKT2, and KKT3. We recently reported the identification of a specific trypanocidal inhibitor of T. brucei CLK1, an amidobenzimidazole, AB1. We now show that chemical inhibition of CLK1 with AB1 impairs inner kinetochore recruitment and compromises cell cycle progression, leading to cell death. Here, we show that KKT2 is a substrate for CLK1 and identify phosphorylation of S508 by CLK1 to be essential for KKT2 function and for kinetochore assembly. Additionally, KKT2 protein kinase activity is required for parasite proliferation but not for assembly of the inner kinetochore complex. We also show that chemical inhibition of the aurora kinase AUK1 does not affect CLK1 phosphorylation of KKT2, indicating that AUK1 and CLK1 are in separate regulatory pathways. We propose that CLK1 is part of a divergent signaling cascade that controls kinetochore function via phosphorylation of the inner kinetochore protein kinase KKT2.

## INTRODUCTION

At the onset of cell division, the accurate distribution of genomic material is crucial for cell survival and development (1). Central to this process are the kinetochores, a centromere macromolecular protein complex that drives chromosome segregation in eukaryotes by connecting chromosomes to microtubules (2). The kinetochore is a large, highly dynamic machine assembled from multiple pathways that are temporally controlled (3). Kinetochores gather on opposite sides of a centromere region of each chromosome where spindle microtubules attach (4). In general, the kinetochore can be thought of as a different set of proteins, assembled by timing blocks. The inner kinetochore, composed of proteins that bind to DNA or centromeric chromatin, is also known as the constitutive centromere-associated network (CCAN) in vertebrates and fungi (5). As a cell enters mitosis, outer kinetochore proteins are assembled on this platform of inner kinetochore proteins, forming the interaction surface for spindle microtubules and allowing chromosome movement (6). Several inner kinetochore components associate with kinetochores throughout the cell cycle, while other inner kinetochore proteins are recruited to the outer surface, specifically in mitosis (7). They provide a landing platform for the spindle assembly checkpoint (SAC) proteins, ensuring the fidelity of chromosome segregation (8).

From yeast to humans, the majority of the CCAN assembly can be subdivided into four discrete units, and their stability depends critically on reciprocal interactions (6). Furthermore, the recruitment of components of the CCAN in these species depends on a specialized centromeric histone H3 variant, the centromere protein A (CENP-A) (9). The fact that some subunits are missing from certain lineages (10) highlights that much remains to be understood about the structural and functional contributions of these four CCAN complexes at the kinetochore. Functional studies indicate that the CCAN plays an active role in the efficient incorporation of CENP-A into centromeric nucleosomes (11), where, afterwards, it is required either for the assembly of further kinetochore components, thereby functioning as a scaffold (2), or the regulation of kinetochore-microtubule dynamics (12).

The emergence of eukaryotes from prokaryotic lineages has involved a significant rise in cellular complexity (13). Research on kinetochores has provided a picture of the essential organization of kinetochores across species. However, the functionality and dynamic organization of the layers that made the kinetochore in some early branch organisms, such as the kinetoplastids, remain unclear (14). This is the case of Trypanosoma brucei, the causative agent of human African trypanosomiasis (HAT), whose kinetochore assembles from a repertoire of unique proteins very divergent from other organisms (15). To date, a trypanosomatid inner kinetochore that contains 24 unique proteins (KKT1 to -23 and KKT25) has been identified (15, 16). Within this group, two proteins with protein kinase domains (KKT2 and -3) are constitutively localized to centromeres throughout the cell cycle, most likely acting as functional orthologues of the eukaryotic CCAN proteins (15, 16). In addition, this parasite has a set of KKT-interacting proteins (KKIP1 to -12) that are related to outer kinetochore proteins Ndc80 and Nuf2 (17) and a cohort of proteins localized to the nucleus during interphase and to the spindle during mitosis (NuSAPs) involved in regulating spindle dynamics and chromosome segregation (18).

Apart from KKT2 and KKT3, the T. brucei kinetochore contains two other protein kinases, CLK1 (KKT10) and CLK2 (KKT19) (15, 19). Previous studies have shown that CLK1 is essential for survival in the bloodstream form of this parasite (20, 21). As part of a drug discovery campaign, we recently identified the amidobenzimidazole AB1 as a trypanocidal covalent inhibitor of T. brucei CLK1. Detailed mode-of-action and target validation studies indicate that CLK1 is the main target of AB, which binds specifically to C215 residue at the hinge domain (22). Treatment of the bloodstream form with AB1 caused nuclear enlargement during metaphase concomitant with a G_2_/M cell cycle arrest. Furthermore, we demonstrated that CLK1 inhibition impaired nuclear KKT2 distribution (22), suggesting that CLK1 has a role in kinetochore assembly or regulation. In the insect procyclic form, KKT4 and KKT7 phosphorylation has been shown to depend on KKT10/19, and the localization of KKT10/19 is tightly controlled to regulate the metaphase-to-anaphase transition (19). Given the clinical importance of T. brucei bloodstream forms for drug intervention and the advantage of using a chemical tool to study the kinetochore regulation, here we demonstrate that CLK1 phosphorylates KKT2 at S508 during early metaphase, and its inhibition affects the posterior recruitment of inner kinetochore components affecting chromosome segregation in a pathway that is independent of aurora kinase B.

## RESULTS

### CLK1 inhibitor AB1 disrupts kinetochore dynamics in bloodstream-form T. brucei.

**FIG 1:**
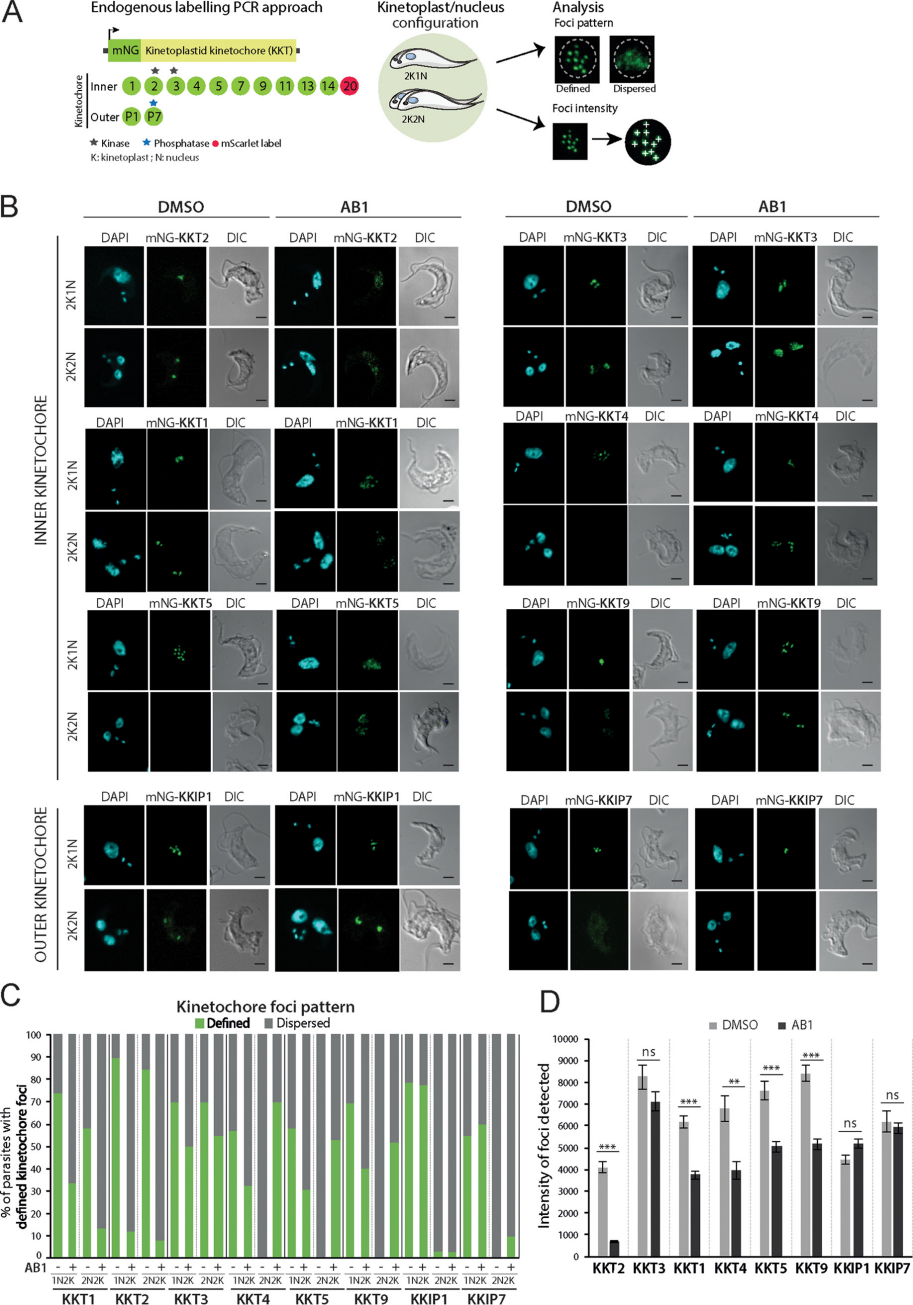
CLK1 inhibition impairs inner kinetochore dynamics. (A) Scheme of the kinetochore assessment workflow by immunofluorescence. A representative cohort of kinetochore components was endogenously labeled with mNeonGreen (mNG) in T. brucei bloodstream forms. Fixed parasites in metaphase or anaphase were considered for analysis of kinetochore pattern and intensity. (B) Localization of inner (top) and outer (lower) kinetochore core components after CLK1 inhibition by AB1. Parasites were incubated or not for 6 h with 5× EC_50_ AB1. Representative fluorescence microscopy micrographs show bloodstream-form parasites endogenously expressing N-terminal mNeonGreen (mNG)-tagged kinetochore proteins. Cells with 2K1N and 2K2N kinetoplast/nucleus configuration are shown. Cells were counterstained with DAPI to visualize DNA (cyan). The right panel shows the corresponding Nomarsky (differential interference contrast [DIC]) images. Scale bar, 2 μm. (C) Percentage of cells in metaphase (1N2K) and anaphase (2N2K) showing a defined kinetochore localization before and after AB1 treatment as described for panel A (*n* > 100 cells in each stage). (D) Intensity of KKT foci detected before (DMSO) and after AB1 treatment. The data represent 75% of total focus intensity (*n* = 80 kinetochores under each condition). Error bars, standard errors of the means (SEM); **, *P* < 0.01, ***, *P* < 0.001; ns, not significant. (Mann-Whitney U test).

Given the importance of kinetochore movement during metaphase in eukaryotes (23), we assessed the impact of T. brucei CLK1 (TbCLK1) activity on kinetochore dynamics using AB1 as a chemical tool. The expression and localization of kinetochore proteins, labeled with mNeonGreen (mNG), were assessed by confocal microscopy in the bloodstream form of the parasite (Fig. 1A). Similar to previous observations in procyclic form cells (15, 17), we observed different kinetochore timings and patterns of expression throughout the cell cycle. By using the kinetoplast (K) and nucleus (N) configuration to define each cell cycle stage (24), we observed that KKT2 and KKT3 are constitutively expressed until anaphase. KKT1 and KKIP1 gradually load from S phase onwards until the end of mitosis, while KKT4 and KKT5 expression is restricted to metaphase. Furthermore, KKT9 and KKIP7 expression diminish during anaphase, suggesting both proteins are acting as scaffolds for the recruitment of multiple other components. Treatment with 5× the 50% effective concentration (EC_50_) of AB1 for 6 h caused dispersal, to various degrees, for KKT1, KKT2, KKT5, KKT9, KKT13, KKT14, and KKT20 from the defined foci of the kinetochore within the nucleus, while KKT3, KKT7, KKT11, KKIP1, and KKIP7 remained in distinct foci (Fig. 1B and C; see also Fig. S1A in the supplemental material). Automated focus detection using subpixel precise single-particle localization combined with image segmentation (25) and intensity quantification (26) determined that there was a significant reduction in focus intensity for KKT1, KKT2, KKT4, KKT5, and KKT9 but not KKT3 (Fig. 1D and Fig. S1B and C). No degradation of these proteins was observed after treatment (Fig. S1D). These results suggest that although KKT2 and KKT3 are centromere-anchored proteins (15), they respond differently to CLK1 inhibition, and that TbCLK1 is a critical regulator of inner kinetochore component dynamics.

10.1128/mBio.00687-21.1 FIG S1 Inner kinetochore core components localization after CLK1 inhibition. (A) Localization of inner kinetochore core components KKT7, KKT11, KKT13, KKT14, and KKT20 after CLK1 inhibition by AB1. Representative fluorescence microscopy micrographs show bloodstream-form parasites expressing N-terminal mNeonGreen (mNG)-tagged KKTs. For KKT20, parasites express N-terminal mScarlet (mS)-tagged protein. Cells with 2K1N and 2K2N kinetoplast/nucleus configurations are shown. Cells were counterstained with DAPI to visualize DNA (cyan). Scale bar, 2 μm. (B) Graphic representation of strategy used for automated identification of kinetochore and background regions and quantification of fluorescence at kinetochore foci. The region of parasite body and nucleus is masked in white, and the region of interest (ROI) quantified for the kinetochore is highlighted with arrows. In this case, KKT2 focus detection in untreated cells was used as an example. (C) The distribution of kinetochore foci, defined as fluorescence intensities, before and after treatment with AB1. Minima of 60 kinetochore foci were measured for each condition; individual points are shown as grey dots. Medians (green) and interquartile ranges (IQR) are shown. **, *P* < 0.01; ***, *P* < 0.001. ns, not significant (Mann-Whitney U test). (D) Western blots comparing total protein levels of KKT proteins after 6 h of treatment with AB1 at 5× EC_50_. EF-1α expression was used as the loading control. Download FIG S1, TIF file, 1.9 MB. Copyright © 2021 Saldivia et al. 2021 Saldivia et al. https://creativecommons.org/licenses/by/4.0/ This content is distributed under the terms of the Creative Commons Attribution 4.0 International license.

### CLK1 phosphorylates KKT2 at position S508.

KKT2 and KKT3 protein kinases are likely components of the trypanosome inner kinetochore with functional equivalence to the constitutive centromere-associated network (CCAN), a canonical component of the eukaryotic inner kinetochore (27). Defective KKT2 clustering was also observed after CLK1 RNA interference (RNAi) (22). It has been reported that phosphorylation of kinetochore proteins has critical roles in kinetochore organization and interaction during mitosis in mammals and yeast (28). Indeed, cell cycle-regulated changes in the phosphorylation of T. brucei kinetochore components have been reported recently, where the regulation is coordinated with phosphorylation of essential protein kinases, including CLK1 (19).

**FIG 2:**
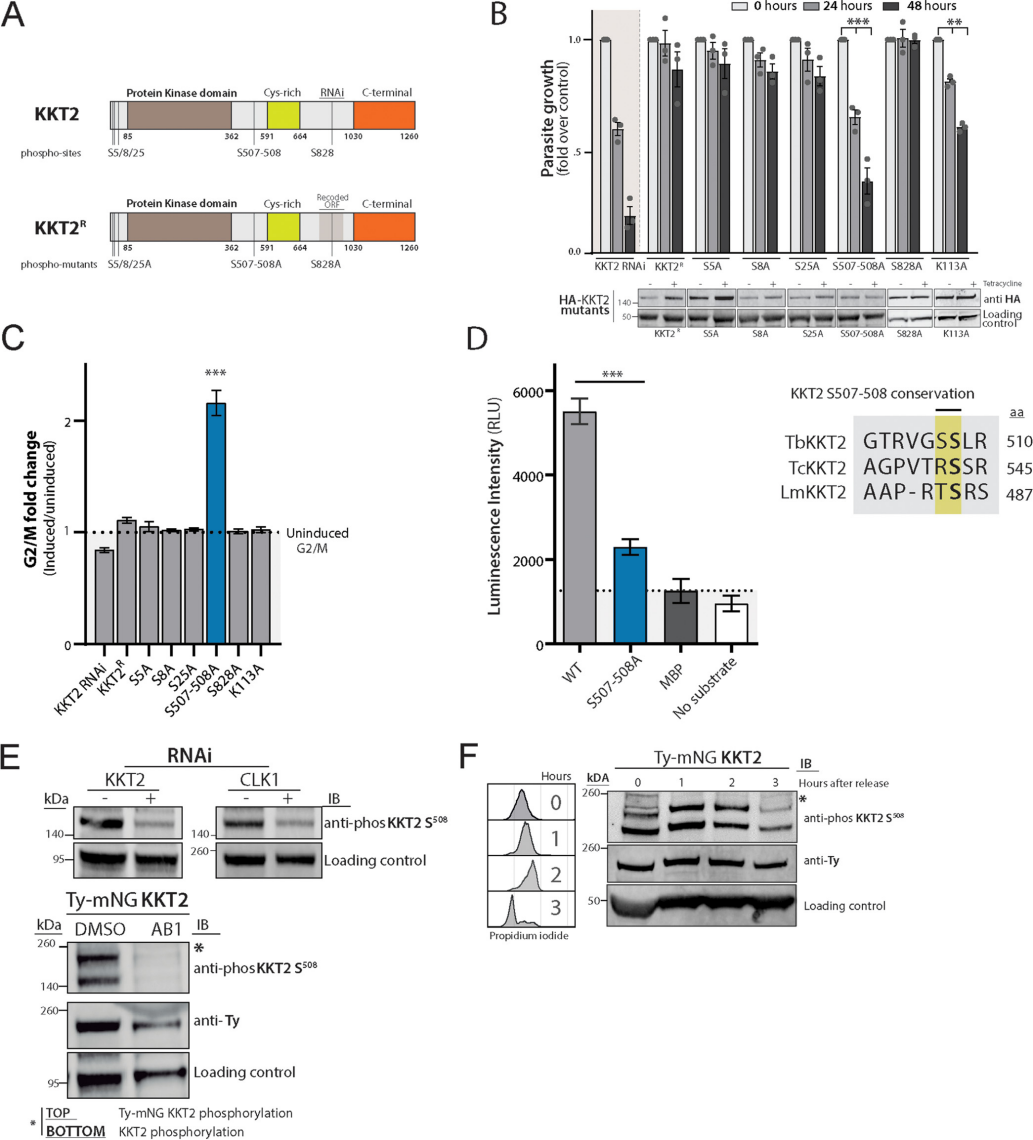
CLK1 regulates KKT2 function by phosphorylation of S508. (A) Schematic representation showing known KKT2 phosphosites and phosphomutants. (B) *In vitro* growth profile of KKT2 RNAi, KKT2^R^, and KKT2^R^ phosphomutants and active-site mutant. Bars show cumulative fold over uninduced control counts over time following tetracycline induction of cell lines in culture. Error bars represent means ± SEM from three replicates; *P* values were calculated using a two-tailed Student’s *t* test: **, *P* < 0.01; ***, *P* < 0.001. (Lower) Western blot of HA-KKT2 mutants. The expression of KKT2 phosphomutants mutants was detected using an anti-HA antibody. EF-1 alpha protein expression was used as the loading control. (C) Cell cycle profile of KKT2 RNAi, KKT2^R^, and KKT2^R^ phosphomutants. Bars showing G_2_/M ratio over the uninduced control following tetracycline induction of cell lines in culture. Error bars represent SEM from 3 replicates. *P* values were calculated using a two-tailed Student’s *t* test: ***, *P* < 0.001. (D) Recombinant CLK1 (rCLK1) phosphorylates recombinant KKT2 *in vitro*. Recombinant fragment of KKT2 including S^507-S508^ (KKT2^486-536^) was used as the substrate for rCLK1 by ADP-Glo kinase assay. The same fragment but including an S^507A-S508A^ mutation (blue) was used as a control. Phosphorylation of maltose binding protein (MBP) and rCLK1 autophosphorylation (no substrate) was included as a control. Error bars, SEM (*n* = 3); ***, *P* < 0.001 (two-tailed Student's *t* test). Conservation of amino acids surrounding KKT2 S507-508 in T. brucei (tb), T. cruzi (tc), and L. mexicana (lm) is shown to the right. (E) Specificity of KKT2 S^508^ phosphospecific antibody. (Top) CLK1 and KKT2 RNAi were induced in 2T1 parasites for 24 h. KKT2 phosphorylation was analyzed by Western blotting using KKT2 S^508^ phosphospecific antibody. (Bottom) Phosphorylation of KKT2 S^508^ and Ty-mNG KKT2 S^508^ after 18 h of treatment with 5× EC_50_ AB1. EF-1 alpha protein expression was used as the loading control. (F) KKT2 S^508^ phosphorylation during the cell cycle. Cells expressing Ty-mNG-tagged KKT2 were synchronized in late S phase by incubating with 10 μM hydroxyurea for 6 h and released. After release, cells were collected after 0, 1, 2, or 3 h, and KKT2 S^508^ phosphorylation was analyzed by Western blotting. Cell cycle progression was assessed by flow cytometry (left) by staining with propidium iodide. Data are representative of two biological replicates.

We speculated that KKT2 provides a platform on which the kinetochore multiprotein complex assembles and that phosphorylation orchestrates this process. To address whether KKT2 is a CLK1 substrate, we first analyzed mobility shifts of phosphorylated forms of KKT2 and KKT3 using Phos-tag gel electrophoresis (29). A low-mobility, nonphosphorylated form of KKT2 was detected after treatment with AB1 or after CLK1 depletion by RNAi, while KKT3 remained unaffected (Fig. S2). Six phosphorylation sites have been identified in KKT2 (S^5^, S^8^, S^25^, S^507^, S^508^, and S^828^) (30), and we tested if these are important for KKT2 function by generating a KKT2 RNAi line (Fig. S3) with a recoded hemagglutinin (HA)-tagged version of KKT2 integrated into the tubulin locus. This constitutively expressed KKT2 (*KKT2^R^*) is not susceptible to RNAi-mediated degradation, and *KKT2^R^* complements the loss of function of KKT2 48 h after RNAi induction (Fig. 2A). Replacement of Ser for Ala in KKT2 at positions S^5^, S^8^, S^25^, and S^828^ resulted in complementation of KKT2 function when expressed in the RNAi line. In contrast, dual replacement of the KKT2 phosphorylation sites S^507^ and S^508^ with Ala (KKT2^S507A-S508A^) failed to complement depletion of the wild-type KKT2 with respect to parasite growth (Fig. 2B) or cell cycle progression after 48 h of induction (Fig. 2C). The efficacy of RNAi knockdown of the endogenous *KKT2* alleles was retained in these derivative cell lines (Fig. S3A, B, and C), demonstrating that the complementation effects were imparted by the recoded alleles. KKT2^S507A-S508A^ had good expression levels in the cell (Fig. 2B, lower) but was mislocalized (Fig. S3D), providing a possible explanation for the phenotype observed. These defects phenocopy the effect of AB1 and show the importance of the two phosphorylation sites for the function of KKT2. To assess whether protein kinase activity is essential for KKT2 function, an active-site mutant was generated in KKT2^R^ (KKT2^K113A^). A significant loss of function was observed after 48 h of induction, indicating protein kinase activity is essential for KKT2 function but not for regulating cell cycle progression (Fig. 2B and C).

10.1128/mBio.00687-21.2 FIG S2 KKT2 phosphorylation is affected by AB1. KKT2 and KKT3 phosphorylation patterns after AB1 treatment or CLK1 depletion by RNAi are shown. Phosphorylation pattern of endogenously tagged Ty-mNG KKT2 and Ty-mNG KKT3 cell lines treated or not with 5× EC_50_ AB1 for 6 h or after 24 h of CLK1 RNAi induction is shown. Protein samples were collected and resolved using Phos-tag technologies. Download FIG S2, TIF file, 0.4 MB. Copyright © 2021 Saldivia et al. 2021 Saldivia et al. https://creativecommons.org/licenses/by/4.0/ This content is distributed under the terms of the Creative Commons Attribution 4.0 International license.

10.1128/mBio.00687-21.3 FIG S3 Verification of endogenous *KKT2* RNAi penetrance throughout strain generation. (A) Schematic of strain derivatives for selected *KKT2* RNAi and recoded addback strains. (B) Schematic depicting key genetic features of endogenous *KKT2* and recoded *KKT2^R^* allowing specific RT-qPCR analysis of endogenous *KKT2* mRNA levels. (C) Results of relative quantitation of endogenous *KKT2* mRNA levels in denoted cell lines by RT-qPCR. Bars and error bars denote mean ± range, *n* = 4; values indicate *P* value results of *t* tests comparing induced versus noninduced samples (D) Representative fluorescence microscopy micrographs showing partial mislocalization of recoded phosphomutant KKT2^S507-508A^, analyzed with anti-HA antibody. Cells with 2K1N and 2K2N kinetoplast/nucleus configuration are shown. Cells were counterstained with DAPI to visualize DNA (cyan). Scale bar, 2 μm. (E) Residue specificity of KKT2 phosphoantibody in recoded KKT2 mutants. Note the absence of signal in the KKT2^S507-508A^ mutant (arrow). Endogenous mutant expression was analyzed by using anti-HA antibody. Download FIG S3, TIF file, 0.6 MB. Copyright © 2021 Saldivia et al. 2021 Saldivia et al. https://creativecommons.org/licenses/by/4.0/ This content is distributed under the terms of the Creative Commons Attribution 4.0 International license.

To address whether CLK1 phosphorylates KKT2 directly at S^507-508^ residues, we expressed a recombinant peptide (amino acids [aa] 486 to 536) of KKT2, including mutations of S507 and S508 residues. We demonstrated that recombinant CLK1 could phosphorylate recombinant KKT2 *in vitro* at positions S507 and S508 (Fig. 2D). Given the conservation of KKT2 S^508^ in kinetoplastids, we then raised a phosphospecific antibody against KKT2^S508^ to monitor KKT2 phosphorylation through the T. brucei cell cycle and after treatment with AB1. The antibody specifically recognizes phosphorylation of KKT2^S508^, as phosphorylated KKT2^S508^ was depleted following *KKT2* or *CLK1* RNAi (Fig. 2E, upper) or after treatment with AB1 in a cell line where KKT2 was endogenously tagged with Ty and mNG (Fig. 2E, lower; both endogenous KKT2 and Ty-mNG KKT2 are detected). In addition, the KKT2 phosphoantibody detects phosphorylated KKT2 in all the recoded mutants except the KKT2^S507A-S508A^ double mutant (Fig. S3E). KKT2^S508^ phosphorylation was found to increase in S-phase after hydroxyurea synchronization and progressively decreased toward G_1_ phase (Fig. 2F), in correlation with the recent demonstration of dynamic KKT2 S508 phosphorylation during the cell cycle (31). Together, these data show that KKT2 phosphorylation is downstream of CLK1 in a kinetochore-specific signaling cascade and occurs during early metaphase.

**FIG 3:**
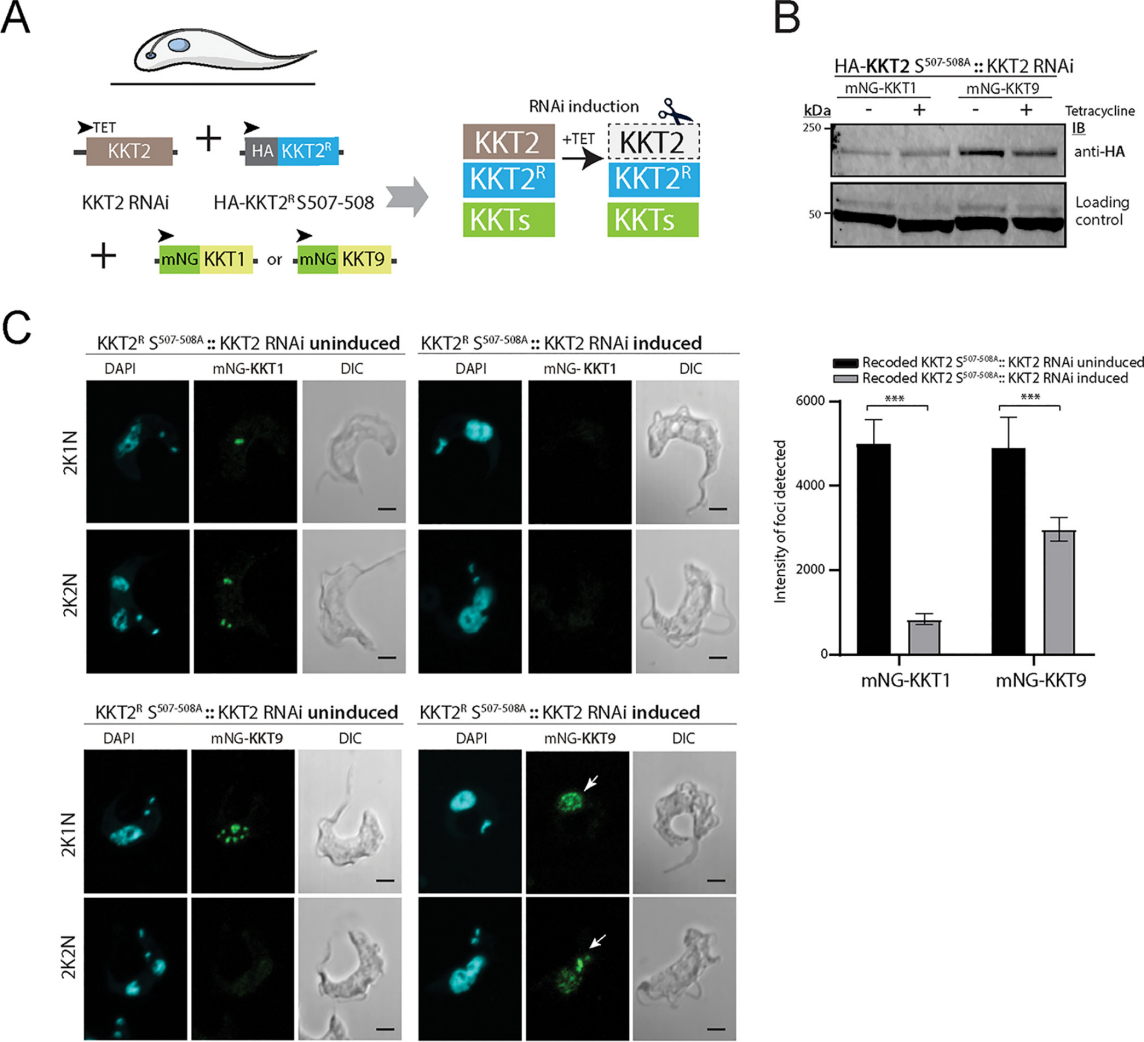
Phosphorylation of KKT2 is required for kinetochore assembly. (A) Schematic representation showing the endogenous labeling of KKT1 or KKT9 in KKT2 recoded S507-508 phosphomutant. (B) Recruitment of KKT1 and KKT9 to the kinetochore is impaired in KKT2^R^ S^507-508A^ mutant. Representative fluorescence microscopy of BSF parasites endogenously expressing KKT1 or KKT9 tagged with mNeonGreen (mNG) at the N terminus. Cells were imaged 48 h after induction of the KKT2^R^ S^507-508A^ mutant. Cells were counterstained with DAPI to visualize DNA (cyan). Scale bar, 2 μm. (C) Regulation of KKT1 and KKT9 in recoded KKT2 S507-508A parasites. (Top) Expression of the recoded HA-KKT2 S507-508A mutant detected by Western blotting using anti-HA antibody. EF-1 alpha protein expression was used as the loading control. (Bottom) Intensity of mNG-KKT1 and mnG-KKT9 foci in recoded KKT2 S507-508A mutants. The data represent 75% of total focus intensity (*n* > 25 kinetochores under each condition). Error bars, SEM; ***, *P* < 0.001 (Mann-Whitney U test).

We next assessed whether KKT2 phosphorylation is required for recruitment of proteins to the trypanosome kinetochore. KKT1 and KKT9 recruitment were impaired in the KKT2^R^S^507A-S508A^::KKT2-induced cell line (Fig. 3A to C) but not the KKT2^R^ K^113A^-induced line (Fig. S4A), underlining the importance of KKT2 phosphorylation by CLK1 for kinetochore assembly. Individual expression of phosphomimetics S^507E^ and S^508E^ impaired KKT1 and KKT9 recruitment but also affected the timing of events during mitosis, with a notable defect in nuclear abscission (Fig. S4B).

10.1128/mBio.00687-21.4 FIG S4 KKT2 impact on KKT1 and KKT9 recruitment. (A) Localization and expression pattern of inner kinetochore proteins KKT1 (left) and KKT9 (right) after expression of recoded catalytically inactive KKT2 R K^113A^. In both mutants, KKT1 and KKT9 proteins were tagged with mNeonGreen (mNG) at the N terminus. Cells with 2K1N and 2K2N kinetoplast/nucleus configuration are shown. Cells were counterstained with DAPI to visualize DNA (cyan). The right panel shows the corresponding Nomarsky (DIC) images. (B) Representative fluorescence microscopy micrographs showing localization of inner kinetochore proteins KKT1 (top) and KKT9 (bottom) after expression of recoded phosphomimetic KKT2R S^507E^ (left) and KKT2R S^508E^ (right). In both mutants, KKT1 and KKT9 proteins were tagged with mNeonGreen (mNG) at the N terminus. Abnormal nuclear shape after induction is shown with white arrows. Cells in metaphase and anaphase are shown. Cells were counterstained with DAPI to visualize DNA (cyan). The right panel shows the corresponding Nomarsky (DIC) images. Scale bar, 2μm. Download FIG S4, TIF file, 0.9 MB. Copyright © 2021 Saldivia et al. 2021 Saldivia et al. https://creativecommons.org/licenses/by/4.0/ This content is distributed under the terms of the Creative Commons Attribution 4.0 International license.

### CLK1 and AUK1 are not part of the same signaling pathway.

**FIG 4:**
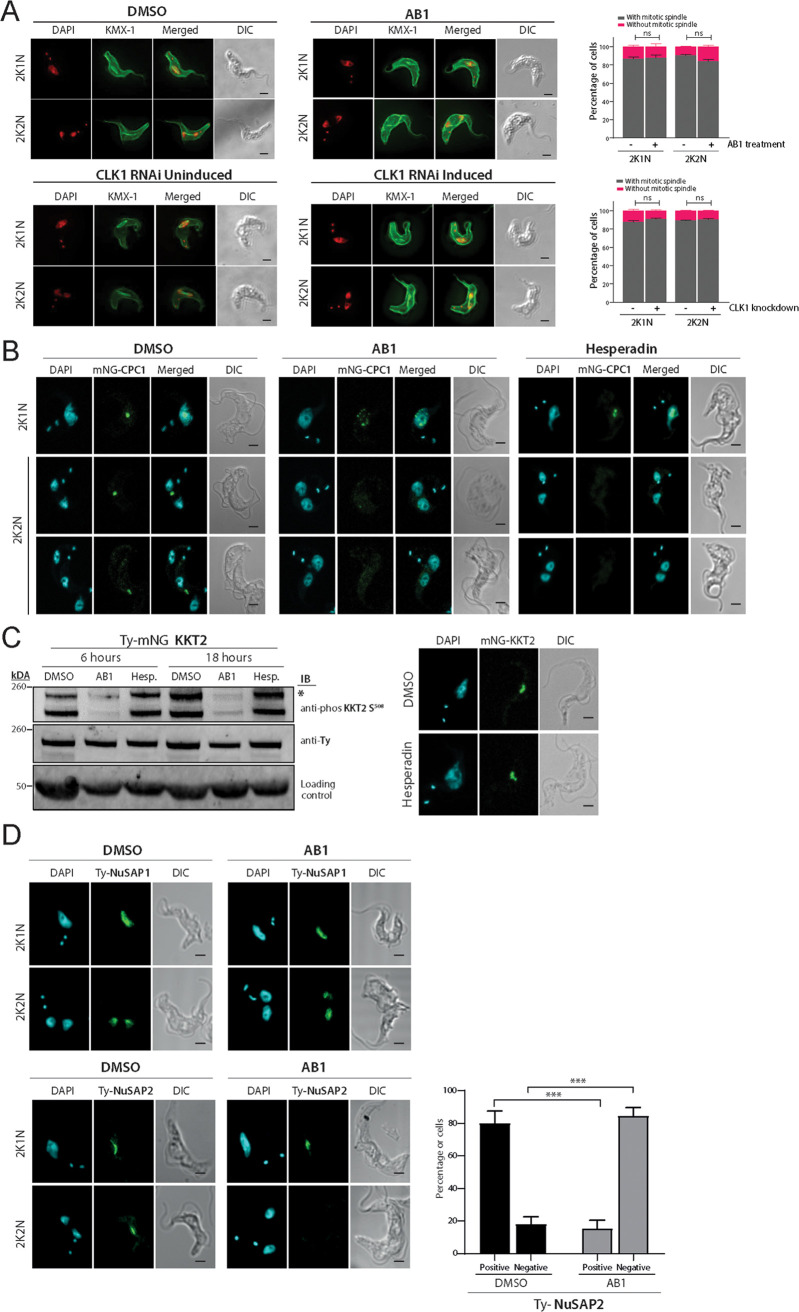
Localization of CPC1 after treatment with AB1 or Hesperadin. (A) Spindle formation after CLK1 inhibition or RNAi knockdown. (Top) Parasites were left untreated or treated for 6 h with 5× EC_50_ AB1 and analyzed by confocal microscopy. (Bottom) CLK1 was depleted by RNAi for 24 h after addition of tetracycline and compared with the uninduced control. Cells with 2K1N and 2K2N kinetoplast/nucleus configuration were analyzed, and spindle formation was assessed by using mouse anti-KMX-1 antibody. Graph bars represent the percentage of cells with (gray) or without (purple) spindle. Error bars, SEM (*n* > 80 cells in each stage). ns, not significant. (B) Ty-mNG-CPC1-expressing parasites were left untreated or treated for 6 h with 5× EC_50_ AB1 or 5× EC_50_ Hesperadin and analyzed by confocal microscopy. Cells in metaphase and anaphase are shown. Cells were counterstained with DAPI to visualize DNA (cyan). The right panel shows the corresponding Nomarsky (DIC) images. (C) Inhibition of aurora kinase (AUK1) does not affect KKT2 S^508^ phosphorylation. (Left) KKT2 S^508^ phosphorylation analyzed by Western blotting in parasites treated or not with 5× EC_50_ and 2× EC_50_ Hesperadin for 6 h and 18 h, respectively. Concurrently, AB1 treatment was used as a positive control under the same conditions. EF-1 alpha protein expression was used as the loading control. (Right) Localization of TY-mNG KKT2 after 6 h of treatment with 5× EC_50_ Hesperadin. Cells in metaphase are shown. Cells were counterstained with DAPI to visualize DNA (cyan). (D) Localization of nucleolar and spindle-associated proteins. Representative fluorescence microscopy micrographs showing localization of nucleolar and spindle-associated proteins 1 (NuSAP1, top) and 2 (NuSAP2, lower) after CLK1 inhibition by AB1. Both proteins were endogenously tagged with Ty at the N terminus. Cells with 2K1N and 2K2N configurations are shown. Cells were counterstained with DAPI to visualize DNA (cyan). The lower right panel shows the quantification in percentage of positive or negative expression of NuSAP2 (*n* = 200) during anaphase in control (DMSO) or treated (AB1) parasites. Error bars, SEM; ***, *P* < 0.001. Two-tailed Student's *t* test. Scale bar, 2 μm.

Faithful chromosome segregation relies on the interaction between chromosomes and dynamic spindle microtubules (32). Furthermore, spindle elongation is important for correct segregation of chromosomes during anaphase (33). To further examine if CLK1 inhibition impairs microtubule spindle dynamics, we observed the expression of the mitotic spindle by staining the parasites with KMX-1 antibody and analyzing the microtubule-associated protein 103 kDa (MAP103) (Fig. S5) (34). This showed that treatment with AB1 does not affect microtubule spindle formation (Fig. 4A). Considering that CLK1 inhibition during metaphase results in an arrest in late anaphase (19, 22), it is likely that the function of CLK1 during cytokinesis is related to either the control of kinetochore-spindle microtubule attachment errors or its interactions with the chromosomal passenger complex (CPC). Of note, it has been reported that T. brucei aurora kinase B has an important role during metaphase-anaphase transition and the initiation of cytokinesis via regulation of the CPC (35–37) and nucleolar and other spindle-associated proteins (NuSAPs) (38).

10.1128/mBio.00687-21.5 FIG S5 MAP103 expression after CLK1 inhibition by AB1. Spindle formation after CLK1 inhibition. Parasites expressing mNG-MAP103 were left untreated or treated for 6 h with 5× AB1 and analyzed by confocal microscopy. Error bars, SEM (*n* > 100 cells in metaphase). Ns, not significant. (Lower) Representative micrograph of each condition. Download FIG S5, TIF file, 0.1 MB. Copyright © 2021 Saldivia et al. 2021 Saldivia et al. https://creativecommons.org/licenses/by/4.0/ This content is distributed under the terms of the Creative Commons Attribution 4.0 International license.

In mammals, kinetochore assembly is enhanced by mitotic phosphorylation of the Dsn1 kinetochore protein by aurora kinase B, generating kinetochores capable of binding microtubules and promoting the interaction between outer and inner kinetochore proteins (39). In T. brucei, aurora kinase B (TbAUK1) plays a crucial role in spindle assembly, chromosome segregation, and cytokinesis initiation (37). Therefore, we asked if CLK1 and AUK1 are part of the same signaling pathway. We showed that treatment with AB1 does not affect spindle formation (Fig. 4A), in contrast to the inhibition of AUK1 (40). AUK1 is a key component of the trypanosome CPC (41). To understand if CPC dynamics are impaired by CLK1 inhibition, we monitored the localization of CPC1 throughout the cell cycle before, after AB1 treatment, and following AUK1 inhibition by Hesperadin (42). After treatment with AB1, CPC1 showed a dispersed nuclear pattern that progressively disappeared after abscission of the nucleus (Fig. 4B, middle). This was different from AUK1 inhibition by Hesperadin, which prevented translocalization of the CPC from the spindle midzone, impairing initiation of cytokinesis (Fig. 4B, right). Finally, we confirmed that AUK1 is not involved in kinetochore assembly, since neither KKT2^S508^ phosphorylation nor KKT2 localization was affected by AUK1 inhibition by Hesperadin (Fig. 4C). In addition, a cohort of divergent spindle-associated proteins has been described that is required for correct chromosome segregation in T. brucei (18). Therefore, we analyzed the subcellular localizations of NuSAP1 and NuSAP2 during the cell cycle after CLK1 inhibition. NuSAP2 expression in the central portion of the spindle after metaphase release was compromised by CLK1 inhibition, while NuSAP1 remained unaffected (Fig. 4D). NuSAP2 is a divergent ASE1/PRC1/MAP65 homolog, a family of proteins that localizes to kinetochore fibers during mitosis, playing an essential role in promoting the G_2_/M transition (43). Considering that NuSAP2 and KKT2 colocalize during interphase and metaphase (18), it is likely that KKT2 regulation by CLK1 influences posterior spindle stability and cytokinesis.

## DISCUSSION

The inner kinetochore complex of T. brucei is unusual in that none of the 24 identified KKT proteins have any sequence identity with CENP proteins of the constitutive centromere-associated network (CCAN) in yeast or vertebrates (15, 16). Four of the KKTs contain protein kinase domains, and here we provide the first evidence of a unique protein kinase signaling pathway that regulates inner kinetochore function in bloodstream-form T. brucei. KKT2 is a multidomain protein, constitutively associated with the centromere during the cell cycle, which contains an N-terminal protein kinase domain, a central domain with a unique zinc finger domain, and a C-terminal divergent polo box domain (PDB) (15). The PBD and the central domain are sufficient for kinetochore localization (44), but it is not clear if KKT2 binds directly to DNA or forms a protein complex at nucleosomes with other KKT proteins. In this study, we show that while KKT2 protein kinase activity is required for growth and replication of bloodstream-form trypanosomes (Fig. 2B), the localization of KKT1 and KKT9 to the kinetochore remained unaffected by the loss of KKT2 protein kinase activity (see Fig. S4A in the supplemental material). These data suggest that KKT2 protein kinase activity is required for a function of the kinetochore that is independent from assembly of its inner complex.

We also show that phosphorylation of the kinetochore, and specifically KKT2, is crucial for kinetochore assembly in bloodstream-form T. brucei. Depletion of the kinetochore protein kinase CLK1 (KKT10) by RNAi, or inhibition with the CLK1 inhibitor AB1, is lethal due to disruption of kinetochore assembly (22). Multiple phosphorylation sites have been identified in KKT2, and a number are cell cycle regulated, including S508 (31), suggesting a regulatory role. While we cannot discount phosphorylation of S507 or other sites as a requirement for kinetochore assembly, we only identified S508 to be essential, indicating that the other known phosphorylation sites cannot compensate for loss of phosphorylation on S508. S508 is located between the Cys-rich central domain and the C-terminal domain, and phosphorylation might contribute to association of KKT2 with chromatin via its DNA binding domain. Indeed, the finding that KKT2^S507A-S508A^ is mislocalized supports this hypothesis, and the fact that the mutant protein can localize to the kinetochore in the presence of wild-type KKT2 suggests that KKT2 is an oligomer and that the WT protein can recruit and retain the mutant protein on the kinetochore. As KKT2 protein kinase activity is not required for assembly of the kinetochore, phosphorylation of S508 seems less likely to regulate the kinase activity of KKT2.

By using chemical and molecular approaches, we demonstrate that phosphorylation of KKT2 in the bloodstream form during metaphase allows the spatial recruitment of inner kinetochore components. We provide evidence that KKT2 is phosphorylated by CLK1, but we cannot formally rule out the possibility of an intermediate kinase being involved. Recently, a study showed that in the procyclic form, CLK1 kinase activity is essential for metaphase-to-anaphase transition, although its expression was dispensable for the recruitment of kinetochore components (19). This difference may be due to cell cycle regulators having different functions in the two developmental stages of T. brucei (45, 46) or because there can be protein turnover differences between life cycle stages (47). Indeed, CLK1 protein expression relative to CLK2 appears higher in the bloodstream trypanosome (22) than the procyclic form (19).

In T. brucei bloodstream forms, we show that KKT2 is a substrate for CLK1. In mammals, CLK protein kinases are found in the cytoplasm and in the nucleus, where they regulate alternative splicing through phosphorylation of serine/arginine-rich domains on splicing factors (48), as occurs with human CLK1 in association with the serine-arginine protein kinase 1 (SRPK1) (49). Human CLKs also activate the abscission checkpoint in human cells by phosphorylating aurora kinase B, most likely acting as upstream regulators (50). The role of CLKs in regulating splicing is conserved across many organisms, including Plasmodium falciparum, where inhibition of P. falciparum CLK1-3 (PfCLK1-3) is lethal to the parasite by preventing the splicing of essential genes (51). In T. brucei, most genes are constitutively transcribed as polycistronic mRNAs that are resolved through trans-splicing (52), but it remains unclear if CLK1 also has a role in that process. It has been proposed that the unique domains structure of T. brucei kinetochore proteins is consistent with the T. brucei kinetochore having a distinct evolutionary origin (15, 44), and the finding of a unique CLK1/KKT2-centered regulation for kinetochore assembly supports that hypothesis.

As with most signaling networks, phosphorylation plays an essential role in the regulation of kinetochore functions, and multiple kinases have been found to regulate kinetochores (53). Key examples are aurora kinase B, MPS1, BUB1, PLK1, and CDK1 (53, 54). From yeast to humans, most of the functions of aurora kinase B require its incorporation into the CPC (55) and its dynamic localization during the cell cycle (54). As a regulator of the kinetochore-microtubule attachment during mitosis, aurora kinase B contributes decisively to two feedback mechanisms, the error correction (EC) and spindle assembly checkpoint (SAC) (56). Furthermore, it promotes the inner and outer kinetochore interactions through phosphorylation of Dsn1 (39, 57, 58), a subunit of the Mis12 inner kinetochore complex, essential for kinetochore assembly (59). The T. brucei aurora kinase B orthologue, TbAUK1, has distinctive roles in metaphase-anaphase transition, ensuring proper spindle assembly, chromosome segregation, and cytokinesis (37, 40). Alongside the parasite CPC, TbAUK1 associates with chromosomes during G_2_/M phase and with kinetochores in metaphase and finally localizes in the spindle midzone in anaphase (41), suggesting a role coordinating kinetochore recruitment and attachment. However, the potential role of this kinase in promoting kinetochore assembly has not yet been established or well separated from its regulatory function on mitosis.

In the T. brucei procyclic form, two kinetochore proteins, KKT4 and KKIP4, localize to the spindle during mitosis (17, 60). Our results suggest that localization/expression of key outer kinetochore proteins remains unaffected after CLK1 inhibition, whereas KKT4, recently described as a microtubule tip-coupling protein (60), remains in anaphase, suggesting end-on interaction defects of microtubules with kinetochores. The role of aurora kinase B in the inner and outer kinetochore interaction in yeast resembles our findings of TbCLK1 functions in the recruitment of inner kinetochore during metaphase. Conversely, our results indicate that both pathways act independently in T. brucei or at least not involving inner plate recruitment through KKT2 phosphorylation; the stability of KKT2 localization further support this hypothesis. Interestingly, inhibition of CLK1 affects CPC localization at metaphase and NuSAP2 during anaphase. Understanding that centromeric localization of CPC is required to correct errors in attachment (61) and NuSAPs stabilize kinetochore microtubules during metaphase (62), it will be possible that, during anaphase onset, CLK1 and TbAUK1 coordinate different layers of regulation of kinetochore microtubule attachment and spindle stabilization. The fact that CLK1 copurifies with TbMlp2 and NuSAP1 provides further support for this (18). Interestingly, NuSAP1 to -4 partially colocalize with KKT2 (a CLK1 substrate) during the cell cycle, and knockdown of NuSAP1 destabilizes the expression of KKT1 but also triggers an unequal nuclear division without affecting spindle assembly (18), similar to our findings with KKT2 phosphomutants. Future experiments are required to determine whether the CLK1-KKT2 axis regulation of inner kinetochore assembly in T. brucei also requires a specific set of NuSAPs.

**FIG 5:**
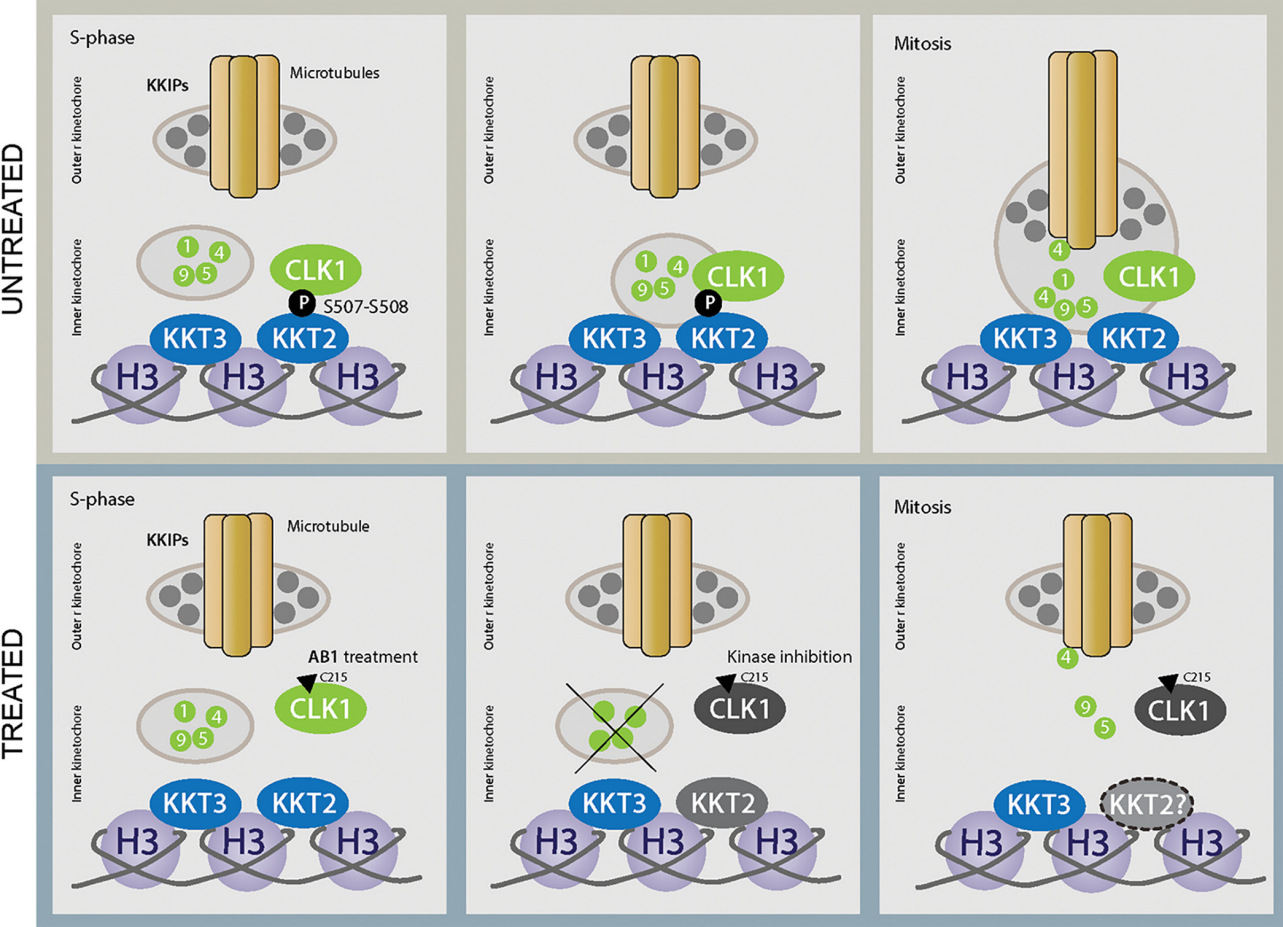
Regulation of kinetochore assembly by CLK1. This schematic diagram summarizes the recruitment defects caused by inhibition of CLK1 by AB1. In untreated cells in metaphase (top), CLK1 phosphorylates KKT2, resulting in recruitment of inner kinetochore components, allowing posterior kinetochore assembly to outer kinetochore components. When CLK1 is inhibited by AB1 (lower, arrowhead), phosphorylation of KKT2 S^508^ is prevented, leading to a failure of recruitment of inner kinetochore components and consequent cell cycle arrest. We hypothesize that KKT2 binding to the centromere is compromised (KKT2?) after CLK1 inhibition (dashed circle). H3, histone H3.

Altogether, we propose a model where CLK1 progressively phosphorylates KKT2 during S phase, allowing the timely spatial recruitment of the rest of the kinetochore proteins and posterior attachment to microtubules (Fig. 5). It is possible that KKT2 is phosphorylated by CLK1 prior to recruitment to the kinetochore, but evidence suggests this would occur during early S phase (32). Inhibition of CLK1 activity with AB1 leads to impaired inner kinetochore assembly and irreversible arrest in M phase, suggesting that this defect cannot be repaired by the parasite’s checkpoint control and implying a dual function of CLK1 at different points during chromosome segregation. Considering the conservation of CLK1 between T. brucei, T. cruzi, and L. mexicana (22), the bioactivity of AB1 against the three trypanosomatids, and the conservation of the KKT2 S508 phosphorylation site in *Leishmania* and T. cruzi, it is quite likely that this signaling pathway is conserved across the trypanosomatids.

## MATERIALS AND METHODS

### Parasites.

All transgenic T. brucei *brucei* parasites used in this study were derived from monomorphic T. brucei *brucei* 2T1 bloodstream forms (63) and were cultured in HMI-11 (HMI-9 [GIBCO] containing 10%, vol/vol, fetal bovine serum [GIBCO], Pen-Strep solution [penicillin at 20 U ml^−1^, streptomycin at 20 mg ml^−1^]) at 37°C and 5% CO_2_ in vented flasks. Selective antibiotics were used: 5 μg ml^−1^ blasticidin or hygromycin and 2.5 μg ml^−1^ phleomycin or G418. RNAi was induced *in vitro* with tetracycline (Sigma-Aldrich) in 70% ethanol at 1 μg ml^−1^. The endogenous Ty, mNeonGreen experiment was performed using the pPOTv6 vector (64). The generation of inducible TbCLK1 and KKT2 RNAi was done as previously described (20).

### Plasmids.

Recoded *KKT2* was synthesized by Dundee Cell Products. The recoded KKT2 sequence (*KKT2^R^*) codes for the same amino acid sequence as *KKT2* but only shares 94.23% nucleotide identity. All segments of identity between *KKT2* and *KKT2^R^* are less than 20 bp long. *KKT2^R^* was inserted into the plasmid pGL2243 using XbaI and BamHI restriction sites, generating pGL2492. This plasmid is designed to constitutively express KKT2 from the tubulin locus, with the addition of a C-terminal 6× HA tag. To express catalytically inactive KKT2 and phosphomutants, the active-site lysine (K^113^) and serine (S^5^, S^8^, S^25^ S^507-S508^, and S^828^) were changed to alanine by mutating pGL2492, carrying the coding sequence for *KKT2*, using site-directed mutagenic PCR. A list of primers is provided in Text S1 in the supplemental material. To generate individual KKT2 recoded mutants, correspondent KKT2^R^ plasmids (described above) were transfected into the KKT2 RNAi cell line. Localization of endogenous KKT1 and KKT9 in KKT2^R^ mutants was assessed by microscopy after transfection of the correspondent mNG-KKT1 or mNG-KKT9 pPOTv6 vector into each recoded cell line.

10.1128/mBio.00687-21.6 TEXT S1 Additional methods not included in the main manuscript. Download Text S1, DOCX file, 0.03 MB. Copyright © 2021 Saldivia et al. 2021 Saldivia et al. https://creativecommons.org/licenses/by/4.0/ This content is distributed under the terms of the Creative Commons Attribution 4.0 International license.

### Immunofluorescence and cell cycle analysis.

Cells treated for 6 h with compounds or dimethyl sulfoxide (DMSO) and were centrifuged at 1,400 × *g* for 10 min before washing twice with *Trypanosoma* dilution buffer (TDB)-glucose at room temperature. Suspensions were centrifuged at 1,000 × *g* for 5 min, pipetted into 6-well microscope slides, and dried at room temperature (RT). Cells were fixed with 25 μl of 2% paraformaldehyde diluted in phosphate-buffered saline (PBS) and incubated at room temperature for 5 min. Cells were washed in PBS to remove paraformaldehyde prior to washing twice more with PBS and permeabilized with 0.05% NP-40 for 10 min. Cells were washed twice in PBS and dried at RT. Mounting medium with 4′,6-diamidino-2-phenylindole (DAPI) was added to each well with a coverslip. Slides were kept at 4°C before viewing using a Zeiss LSM 880 with Airyscan on an Axio Observer.Z1 invert confocal microscope.

Ty-NuSAP1 and Ty-NuSAP2 were detected by indirect immunofluorescence by using a mouse Imprint monoclonal anti-Ty1 antibody (clone BB2). Briefly, cells were harvested by centrifugation at 1,400 × *g* for 10 min at room temperature, washed, and resuspended in TDB-glucose. A total of 2 × 10^5^ cells were dried on slides, fixed in 1% paraformaldehyde (PFA) for 1 h, washed with PBS, blocked with 50% (vol/vol) fetal bovine serum for 30 min, and then incubated with anti-TY (1:800) diluted in 0.5% blocking reagent for 1 h. Alexa-Fluor 488 (anti-mouse) was used as the secondary antibody (Invitrogen). Cells were DAPI stained and visualized using a Zeiss LSM 880 with Airyscan on an Axio Observer.Z1 inverted confocal microscope.

To study spindle formation, wild-type bloodstream forms were treated or not for 6 h with AB1 (5× EC_50_) or CLK1 RNAi cells were treated or not with tetracycline for 24 h. Parasites were harvested by centrifugation at 1,400 × *g* for 10 min and then washed twice with TDB-glucose at room temperature. Samples were fixed for 10 min in 2%, wt/vol, formaldehyde in PBS, followed by 5 min incubation with 1 M Tris, pH 8.5, to quench the fixation. The fixed cells were washed with PBS, suspended in PBS, and adhered to SuperFrost Plus adhesion slides for 15 min. Attached parasites were then permeabilized with methanol at –20°C for 15 min and rehydrated with PBS, followed by incubation with blocking buffer (5% bovine serum albumin, 0.1% Triton X-100 in PBS) for 1 h at room temperature. Cells were immunostained at room temperature for 1 h with KMX-1 antibody to detect the mitotic spindle. After three washes (0.1% Triton X-100 in PBS), samples were incubated for 1 h with an Alexa Fluor 488-conjugated goat anti-mouse IgG (used at 1:300) secondary antibody. Finally, after three more washes, the slides were mounted in ProLong diamond antifade mountant with DAPI and examined by fluorescence microscopy. For analysis, 2K1N and 2K2N populations (*n* = 80) were considered, and statistical significance determined using the Holm-Sidak *t* test, with α = 0.05.

For cell cycle analysis, bloodstream-form T. brucei cell lines were incubated or not for 6 h with AB compounds at a final concentration of 5× the individual EC_50_ for each compound (averaged from viability assays). Control cultures were treated with 0.5 μl DMSO. Cultures were pelleted and cells were collected and washed once in TDB supplemented with 5 mM EDTA and resuspended in 70% methanol. Cells were centrifuged at 1,400 × *g* for 10 min to remove methanol and washed once in TDB with 5 mM EDTA. Cells were resuspended in 1 ml 1× TDB with 5 mM EDTA, 10 μg ml^−1^ propidium iodide, and 10 μl of RNase A. Cell suspensions in 1.5-ml tubes were wrapped in foil to avoid bleaching by light. Cells were incubated for 30 min at 37°C in the dark until fluorescence-activated cell sorting (FACS) analysis. Cells were analyzed for FACS using a Beckman Coulter CyAn ADP flow cytometer (excitation, 535 nm; emission, 617 nm). Cell cycle phase distribution was determined by fluorescence.

Hydroxyurea-induced synchronization of cell lines was obtained by incubating parasites in exponential growth phase with 10 μM hydroxyurea (HU) (Sigma-Aldrich) for 6 h. Removal of HU from the culture medium was achieved by centrifuging cells at 1,400 × *g* for 10 min, washing twice with fresh (drug-free) medium, and resuspending cells in medium lacking HU. Subsequently, samples were collected each hour for posterior cell cycle analysis by propidium iodide staining.

### Protein analysis.

KKT2 and KKT3 phosphorylation profile were analyzed by using a SuperSep Phos-tag precast gel (29) according to the manufacturing protocol. Briefly, Ty-mNG KKT2 and Ty-mNG KKT3 were incubated with 5× AB1 EC_50_ for 18 h and collected for analysis by Western blotting in an EDTA-free radioimmunoprecipitation assay (RIPA) lysis buffer. In parallel, the expression of both proteins was analyzed after 24 h for TbCLK1 RNAi. After electrophoresis, the gel was washed 5 times with 10 mM EDTA transfer buffer to improve transference. The membrane then was transferred to a polyvinylidene difluoride (PVDF) membrane using a 0.1% SDS Tris-glycine transfer buffer at 90 mA overnight at 4°C. The membrane was blocked for 1 h with 10% bovine serum albumin (BSA) and KKT2 and KKT3 phosphorylation pattern was analyzed by using an anti-Ty1 antibody (see Text S1 for details).

Anti-phospho KKT2 S^508^ was raised against a synthetic phosphopeptide antigen C-GTRVGS(pS*)LRPQRE-amide, where pS* represents phosphoserine. The peptide was conjugated to keyhole limpet hemocyanin (KLH) and used to immunize rabbits. Phosphopeptide-reactive rabbit antiserum was first purified by protein A chromatography. Further purification was carried out using immunodepletion by nonphosphopeptide resin chromatography, after which the resulting eluate was chromatographed on a phosphopeptide resin. Anti-antigen antibodies were detected by indirect enzyme-linked immunosorbent assay with unconjugated antigens passively coated on plates, probed with anti-IgG-horseradish peroxidase conjugate, and detected with 2,2′-azinobis(3-ethylbenzthiazolinesulfonic acid) substrate. Posterior antigen specificity was confirmed by Western blotting using KKT2 RNAi and endogenous tagged KKT2 cell lines. Custom antibody was produced by Thermo Fisher Scientific.

For Western blotting, parasites were washed with TDB supplemented with 20 mM glucose. After centrifugation, the samples were resuspended in the RIPA buffer (number 9806S; New England Biolabs) supplemented with protease and phosphatase inhibitors obtained from Promega and Roche Life Science, respectively. All samples were quantified by Bradford protein assay (Bio-Rad), 25 μg of protein was loaded, resolved in a 4 to 20% NuPAGE Bis‐Tris gel (Invitrogen) in NuPAGE morpholinepropanesulfonic acid running buffer, and transferred onto Hybond‐C nitrocellulose membranes (GE Healthcare) at 350 mA for 2 h or, for high-molecular-weight proteins, overnight at 4°C.

After transfer, membranes were washed once in 1× TBST (Tris-buffered saline [TBS], 0.01% Tween 20 [Sigma-Aldrich]) for 10 min and then incubated for 1 h in blocking solution (1× TBST, 5% BSA) or, if required, overnight at 4°C. Next, the membrane was rinsed for 10 min in 1× TBST and placed in blocking buffer containing the required primary antisera for 1 h at room temperature or overnight at 4°C. The membrane was then washed 3 times with TBST and placed in blocking solution containing the appropriate fluorescent secondary antisera for 1 h. A list of antibodies is provided in Text S1.

### General statistics.

All statistical analyses were performed using GraphPad Prism 8 (http://www.graphpad.com/scientific-software/prism/). The appropriate tests were conducted and are detailed in the corresponding figure legends.
