# Supplementary figures and images for: A CLK1-KKT2 Signaling Pathway Regulating Kinetochore Assembly in Trypanosoma brucei
Source: mBio. 2021 Jun 15;12(3):e00687-21. doi: 10.1128/mBio.00687-21 (PMC8262961; doi:10.1128/mBio.00687-21)

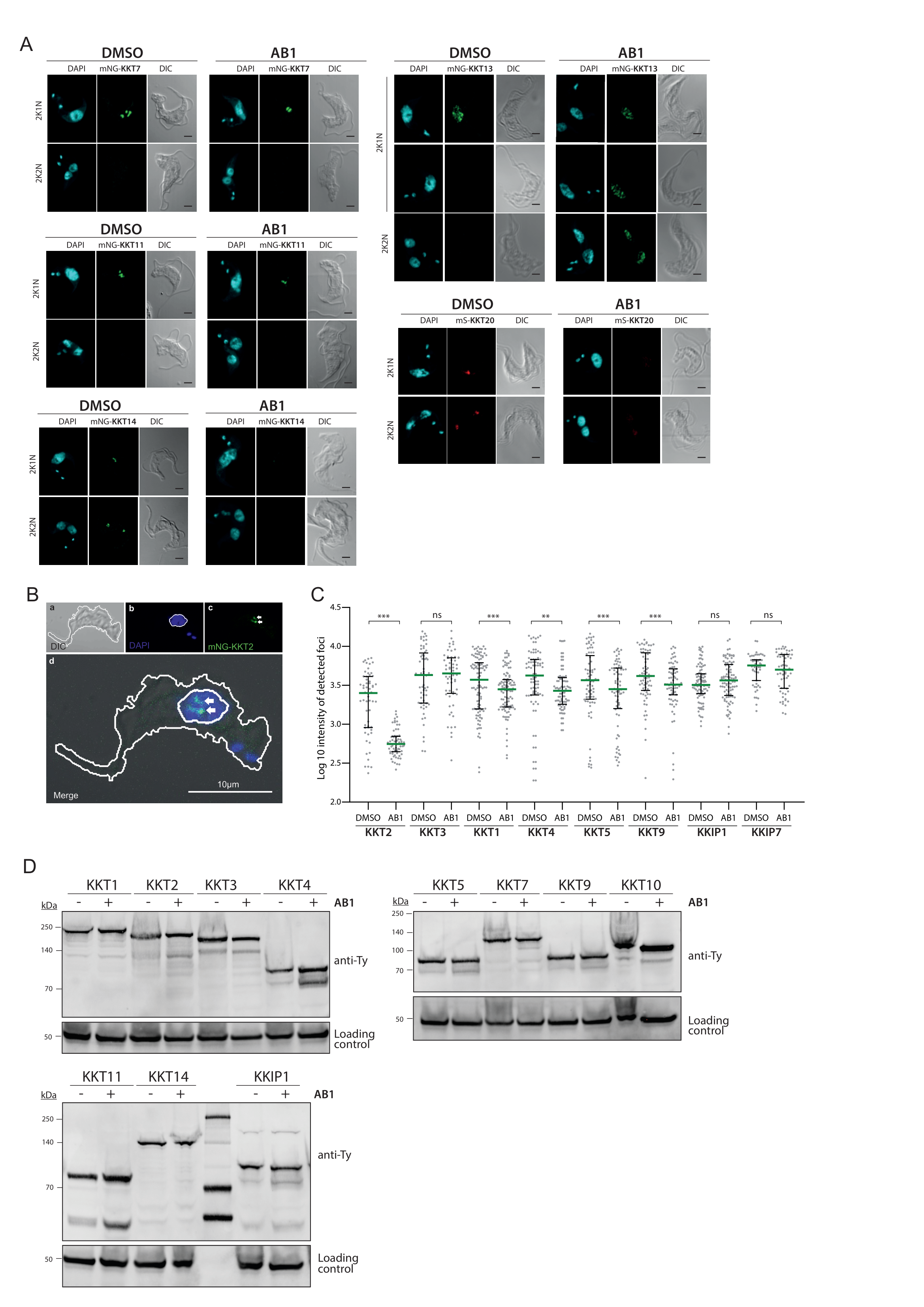

Supplement: FIG S1 [file mbio.00687-21-sf001.tif]

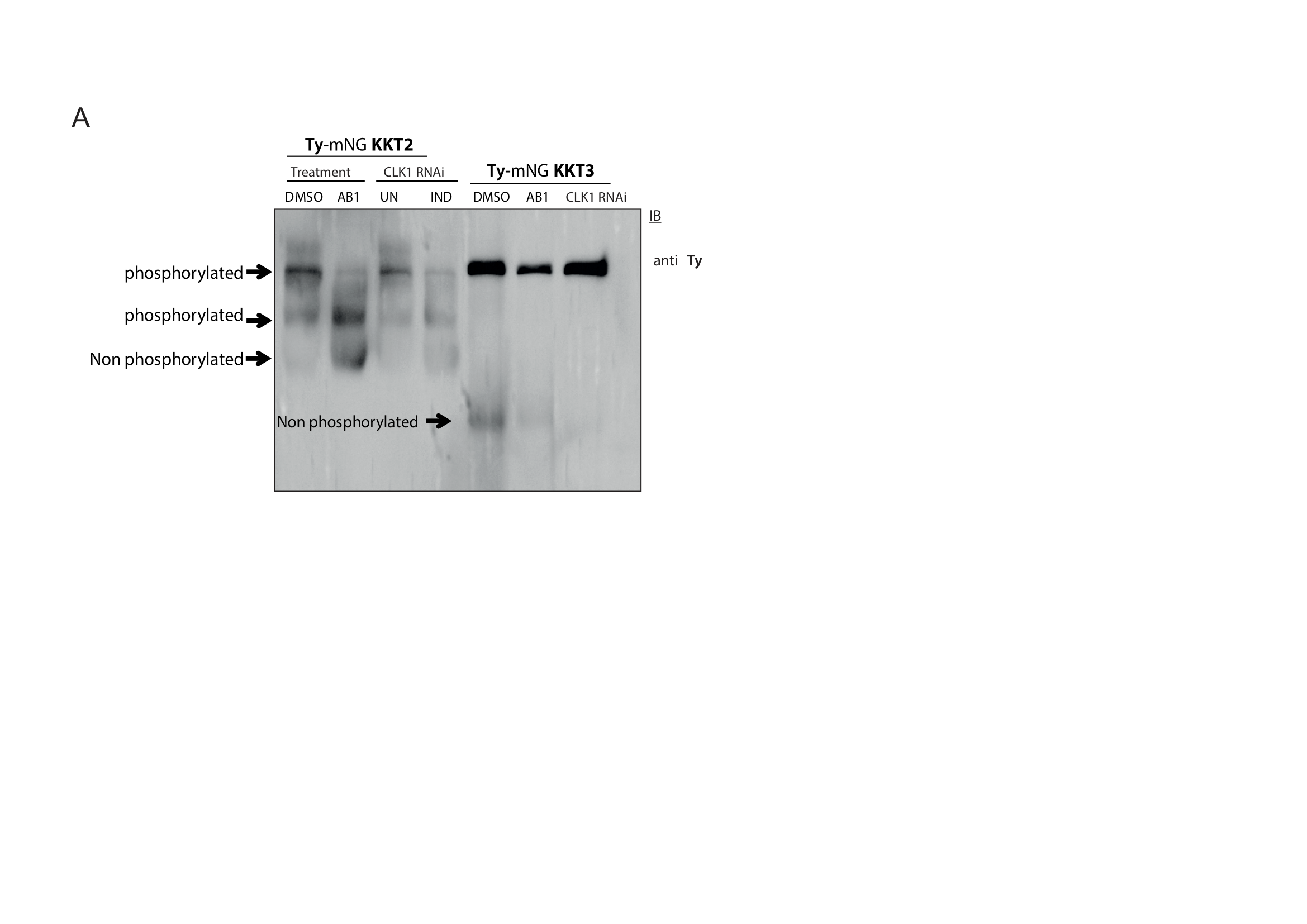

Supplement: FIG S2 [file mbio.00687-21-sf002.tif]

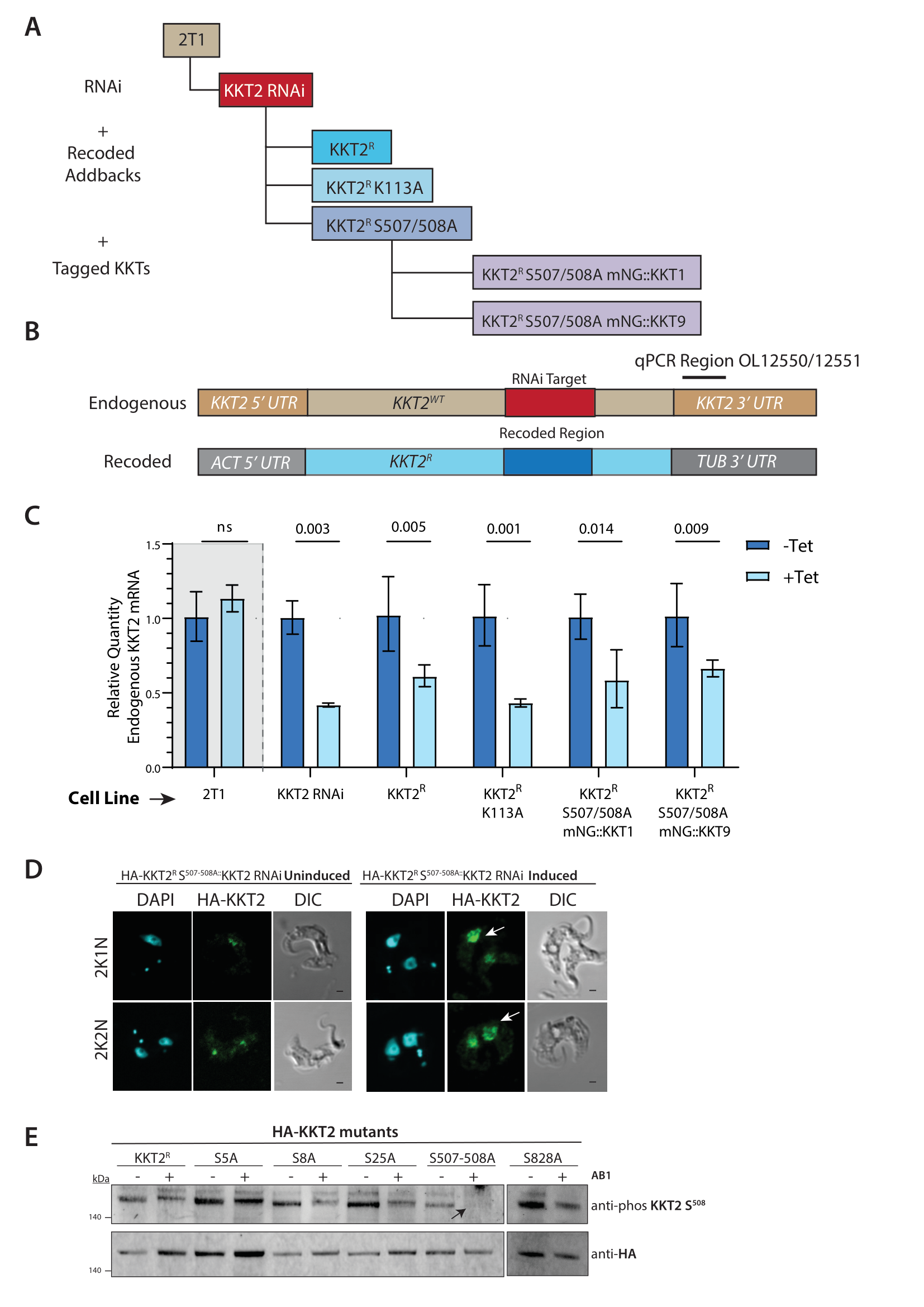

Supplement: FIG S3 [file mbio.00687-21-sf003.tif]

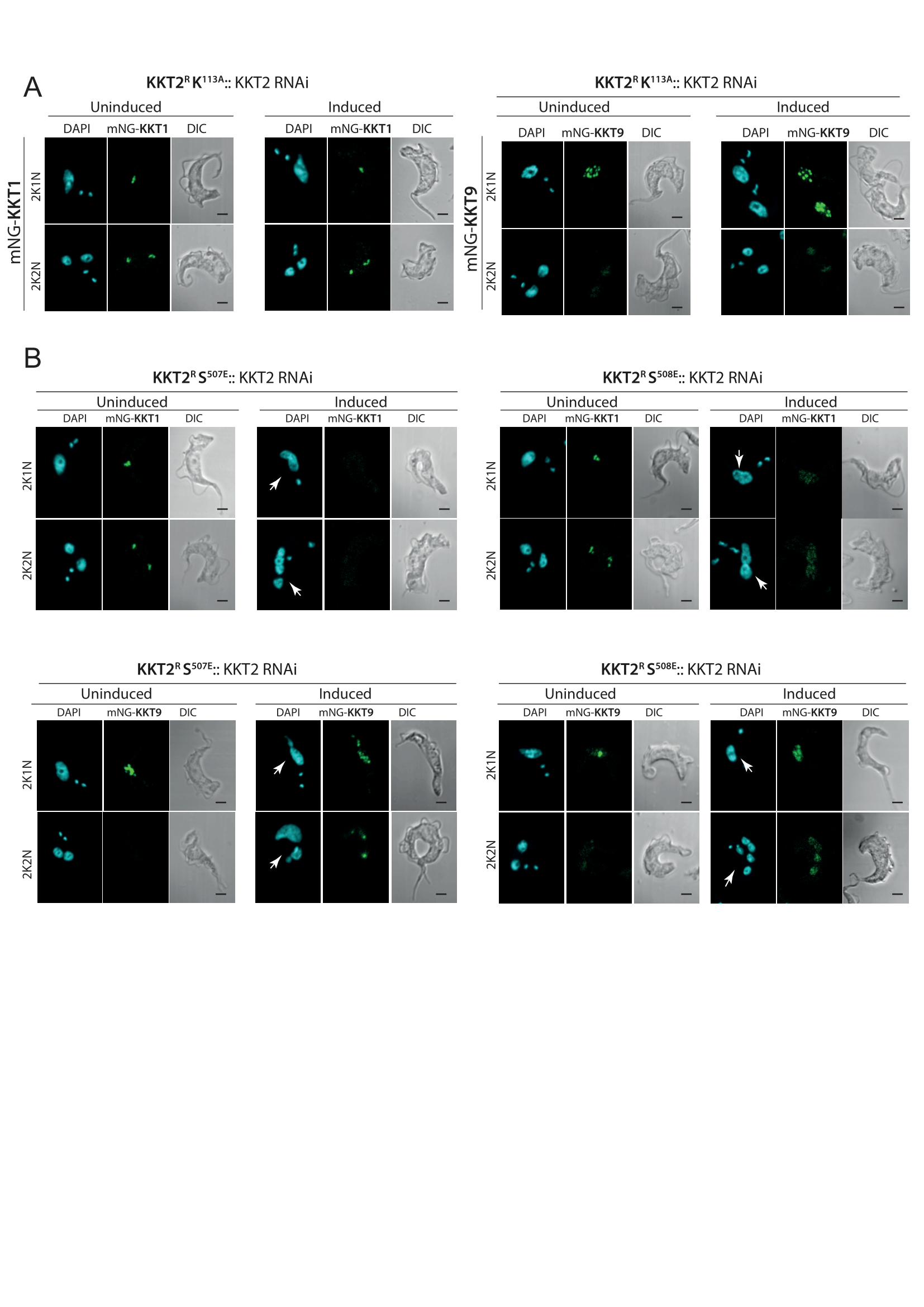

Supplement: FIG S4 [file mbio.00687-21-sf004.tif]

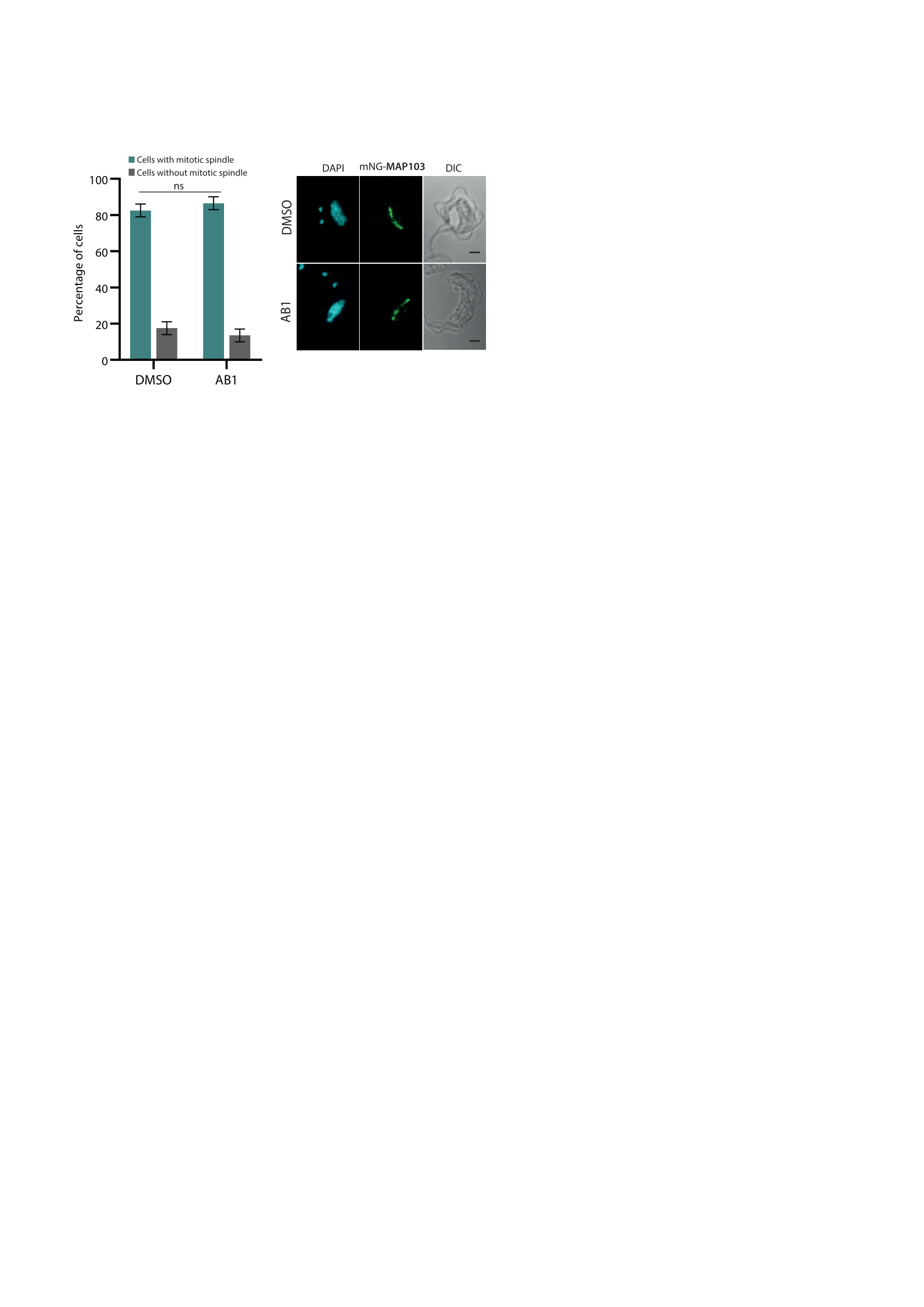

Supplement: FIG S5 [file mbio.00687-21-sf005.tif]
