# Supplementary material for: A CLK1-KKT2 Signaling Pathway Regulating Kinetochore Assembly in Trypanosoma brucei
Source: mBio. 2021 Jun 15;12(3):e00687-21. doi: 10.1128/mBio.00687-21 (PMC8262961; doi:10.1128/mBio.00687-21)
Supplement: TEXT S1 [file mbio.00687-21-s0001.docx]

**Supplementary Methods**

**Recoded Plasmids.** To express catalytically inactive KKT2 and phospho-mutants, the active site lysine (K113) and serine (S5, S8, S507-S508 and S828) were changed to alanine by mutating pGL2492, carrying the coding sequence for KKT2, using site directed mutagenic PCR as follows:

| PRIMER SEQUENCES | MUTATION | PLASMID |
| --- | --- | --- |
| 5’- GTTCAATGTCGCGCCAGCGAGTC | KKT2^R^ **S5A** | pGL2795 |
| 3’- ATTCTAGATATTTTATGGCAGCAAC |  |  |
| 5’- CTCACCAGCGGCGCGTGACCGCG | KKT2^R^ **S8A** | pGL2796 |
| 3’- ACATTGAACATTCTAGATATTTTATGGCAG |  |  |
| 5’- CCCCCGCAGCGCGCTCTCCATGC | KKT2^R^ **S25A** | pGL2797 |
| 3’- CGCGGGGTGCGCTGACTC |  |  |
| 5’- AAGGGTCGGTGCAGCATTAAGGCCGC | KKT2^R^ **S507A-S508A** | pGL2749 |
| 3’- GTTCCACGTTTGGGCTGTTTT |  |  |
| 5’- TAGACAGAATGCATGCGAGCCTTATGCACC | KKT2^R^ **S828A** | pGL2750 |
| 3’- GCTTCGGTTCTGACCCTC |  |  |
| 5’- GTGCGCCTTGGCAGTATCGTCGAAAC | KKT2^R^ **K113A** | pGL2850 |
| 3’- AACTCCCCGCCACTTGAC |  |  |
| 5’- AAGGGTCGGTGAATCATTAAGGCCG | KKT2^R^ **S507E** | pGL2770 |
| 3’- GTTCCACGTTTGGGCTGT |  |  |
| 5’- GGTCGGTTCCGAATTAAGGCCGC | KKT2^R^ **S508E** | pGL2759 |
| 3’- CTTGTTCCACGTTTGGGC |  |  |

**Antibodies**

| **Antibody** | **Obtained from** |
| --- | --- |
| Mouse Imprint Monoclonal **Anti-Ty1** antibody (clone BB2) | Sigma Aldrich |
| Mouse **Anti-HA** (clone 12CA5) | Roche |
| Mouse **Anti-EF1α** Antibody, (clone CBP-KK1) | Merck-Milipore |
| Rabbit **Anti-phospho KKT2 S^508^** | Invitrogen |
| Mouse monoclonal anti-**KMX-1** | Keith Gull laboratory |
| StarBright™ Blue 520 Goat anti-Mouse IgG | BIORAD |
| StarBright™ Blue 700 Goat Anti-Mouse/Anti-Rabbit IgG | BIORAD |

**RT-qPCR for validation of KKT2 RNA Knockdown***.* RNAi inductions were set up to be able to collect 2 x 10^7^ trypanosomes at 24h post tetracycline addition. Total RNA was extracted from these cell pellets using the NEB Monarch RNA Miniprep kit to manufacturer’s instructions. Contaminating gDNA was removed using TURBO DNA-free treatment (Invitrogen). One hundred nanograms of total RNA was then used to prime RT-qPCR reactions set up using Luna Universal One-Step RT-qPCR Kit (NEB), which were amplified and measured using the SYBR and ROX channels of an Applied Biosystems QuantStudio 3 System machine. Oligonucleotides were designed using Primer-BLAST against the 3’ UTR of the KKT2 gene to allow quantification of the WT allele and avoid the RNAi stem-loop RNA and the recoded KKT2 allele’s mRNA. Primer efficiencies were previously verified to be between 95% and 105% using a standard curve analysis prior to relative quantitation experiments. Relative quantitation experiments were performed using the ∆∆ct method with the Tb927.10.12970 (C1) as an endogenous control (1). Samples for comparison were run in technical quadruplicates. No-reverse transcriptase and no-template controls were included on each plate for each sample condition, each in duplicate. Data were analysed in the RQ module of ThermoFisher Cloud to perform the relative quantitation including the T-test option for comparing induced to non-induced samples.

**KKT2 qPCR Primers**

| **Oligo** | **Target** | **Gene ID** | **Sequence** | **Description** | **Efficiency** |
| --- | --- | --- | --- | --- | --- |
| OL12565 | **C1** | Tb927.10.12970 | 5'-TTGTGACGACGAGAGCAAAC | Endogenous control | 100.14% |
| OL12566 |  |  | 3'-GAAGTGGTTGAACGCCAAAT |  |  |
| OL12550 | **KKT2** 3' UTR | Tb927.11.10520 | 5'-CGCTTCTGTGTTCGGGTACT | KKT2 | 96.70% |
| OL12551 |  |  | 3'-AGGTGGTCGGACACTGGATA |  |  |

**Recombinant assays and enzyme purification.** Recombinant full-length CLK1 was produced as described (*22*). For KKT2 protein production, the KKT2 (aa 486 - 536) CDS was cloned in pET24-MBP-TEV vector, generating the plasmid NITD2500. Recombinant expression was carried out by lactose autoinduction in Terrific Broth containing 0.4% glycerol, 0.05% glucose, 0.05% lactose, 0.05% arabinose and buffered by 100 mM sodium phosphate (pH 7.0). In brief, 0.7 L of this media was inoculated at 0.1 OD600 with an overnight Luria Broth culture and shaken at 37 ˚C and 250 rpm for 2.5 hr. Then, temperature was lowered to 18 ˚C and the culture was allowed to grow and induced overnight and harvested 20-24 hr later. Cells are pelleted and stored at -80 ˚C prior to purification. Cell lysis was done by sonication in an ice bath (20 sec ON/OFF, 3 min active sonication at 70-110 watts power) in 40 mL Equilibration Buffer (25 mM HEPES pH 7.5 300 mM NaCl 5% glycerol 0.5 mM TCEP) and the clarified lysate is purified by IMAC on a 5 mL HisTrap column (GE Healthcare). The IMAC elution was further purified by sizing on a 300 mL Superdex 200 prep grade column (GE Healthcare) packed in a 2.6 cm diameter housing. Included volume fractions were pooled and analysed by SDS-PAGE or LC-MS.

To express recombinant KKT2^S507-508A^, plasmid NITD2500 was mutated using site directed mutagenic PCR as follows to give plasmid NITD2501:

| PRIMER SEQUENCES | MUTATION | PLASMID |
| --- | --- | --- |
| 5’- GCGTGTGGGGgcagcaTTGCGCCCGC | KKT2 **S507-508A** | NITD2501 |
| 3´- GTCCCACGCTTAGGCTGT |  |  |

Recombinant CLK1 enzyme activity assays were performed in white 96 well, solid bottom plate (GREINER) by triplicate. The assay buffer contained 40 mM Tris (pH 7.5), 20 mM MgCl2, 0.1mg/ml BSA and 2 mM DTT. As indicated, kinase reaction contains the enzyme CLK1 (3 nM), and 1 micromolar or each indicated substrate. Maltose binding protein (MBP, Abcam ab219252) and DMSO were added as control of background or autophosphorylation respectively. ATP (10 µM) was added to initiate the reaction. After 25 min reaction at room temperature, the ADP-Glo reagent and detection solution was added following the technical manual of ADP-GloTM kinase assay kit (Promega). The luminescence was measured on CLARIOstar BMG LABTECH microplate reader.

**Kinetochore foci intensity capture and analysis.** Cells were imaged using a Zeiss LSM 880 with Airyscan on an Axio Observer.Z1 inverted confocal microscope. A Plan-Apochromat 63x/1.4 oil objective lens was used to image 476 x 476 70nm pixels with a photomultiplier tube and 16x averaging at 38s/frame. DAPI and mNeonGreen excitation were from 405 and 488nm lasers with detection wavelengths 416-479nm and 491-589nm respectively. For measurement of kinetochore foci intensity, three channel image stacks of mNeonGreen labelled kinetochore components in fixed trypanosomes were analyzed using bespoke Matlab software (available here <https://github.com/awollman>). Blue, nuclear stained images were first segmented by thresholding using Otsu’s method and applying a series of morphological transformations to remove holes and any objects smaller than 300-pixel area. This allowed the nucleus to be segmented and removed any detected mitochondria, also stained by DAPI. The whole cell was then segmented from the DIC image, using edge detection and similar morphological transformation, combined with watershedding, using the nuclear mask as ‘seeds’ for each cell. Finally, bright foci were detected in the mNeonGreen image using spot detection software optimized for detecting and characterizing low intensity foci in noisy cellular environments (2, 3). In brief, candidate foci were detected by thresholding and Gaussian masking, before their local background corrected intensity was determined and accepted if above a threshold based on the standard deviation of local pixel noise. Each detected cell was assigned a tracking number and foci categorized into each cell. This allowed for manual assignment into cell cycle stage. Fluorescent foci intensity was maintained in the linear range by optimizing the Imaging conditions using untreated cells to make the best use of the dynamic range of the detector while avoiding saturation. Microscope settings were kept constant between samples, and no saturation was detected, ensuring foci remained in the linear intensity regime.

**Recombinant assays and enzyme purification.** Recombinant full-length CLK1 was produced as described (4) For KKT2 protein production, the KKT2 (aa 486 - 536) CDS was cloned in pET24-MBP-TEV vector, generating the plasmid NITD2500. Recombinant expression was carried out by lactose autoinduction in Terrific Broth containing 0.4% glycerol, 0.05% glucose, 0.05% lactose, 0.05% arabinose and buffered by 100 mM sodium phosphate (pH 7.0). In brief, 0.7 L of this media was inoculated at 0.1 OD600 with an overnight Luria Broth culture and shaken at 37 ˚C and 250 rpm for 2.5 hr. Then, temperature was lowered to 18 ˚C and the culture was allowed to grow and induced overnight and harvested 20-24 hr later. Cells are pelleted and stored at -80 ˚C prior to purification. Cell lysis was done by sonication in an ice bath (20 sec ON/OFF, 3 min active sonication at 70-110 watts power) in 40 mL Equilibration Buffer (25 mM HEPES pH 7.5 300 mM NaCl 5% glycerol 0.5 mM TCEP) and the clarified lysate is purified by IMAC on a 5 mL HisTrap column (GE Healthcare). The IMAC elution was further purified by sizing on a 300 mL Superdex 200 prep grade column (GE Healthcare) packed in a 2.6 cm diameter housing. Included volume fractions were pooled and analysed by SDS-PAGE or LC-MS.

To express recombinant KKT2^S507-508A^, plasmid NITD2500 was mutated using site directed mutagenic PCR as follows to give plasmid NITD2501:

| PRIMER SEQUENCES | MUTATION | PLASMID |
| --- | --- | --- |
| 5’- GCGTGTGGGGgcagcaTTGCGCCCGC | KKT2 **S507-508A** | NITD2501 |
| 3´- GTCCCACGCTTAGGCTGT |  |  |

Recombinant CLK1 enzyme activity assays were performed in white 96 well, solid bottom plate (GREINER) by triplicate. The assay buffer contained 40 mM Tris (pH 7.5), 20 mM MgCl2, 0.1mg/ml BSA and 2 mM DTT. As indicated, kinase reaction contains the enzyme CLK1 (3 nM), and 1 micromolar or each indicated substrate. Maltose binding protein (MBP, Abcam ab219252) and DMSO were added as control of background or autophosphorylation respectively. ATP (10 µM) was added to initiate the reaction. After 25 min reaction at room temperature, the ADP-Glo reagent and detection solution was added following the technical manual of ADP-GloTM kinase assay kit (Promega). The luminescence was measured on CLARIOstar BMG LABTECH microplate reader.

1. S. Kabani et al., Genome-wide expression profiling of in vivo-derived bloodstream parasite stages and dynamic analysis of mRNA alterations during synchronous differentiation in *Trypanosoma brucei*. BMC Genomics. **10**, 427 (2009).

2. J. Wollman et al., Transcription factor clusters regulate genes in eukaryotic cells. Elife. **6** (2017), doi:10.7554/eLife.27451.

3. H. Miller, Z. Zhou, A. J. M. Wollman, M. C. Leake, Superresolution imaging of single DNA molecules using stochastic photoblinking of minor groove and intercalating dyes. Methods. **88**, 81–88 (2015).

4. M. Saldivia et al., Targeting the trypanosome kinetochore with CLK1 protein kinase inhibitors. Nat. Microbiol. **5**, 1207–1216 (2020).
